# Attenuating effect of standardized fruit extract of *punica granatum L* in rat model of tibial and sural nerve transection induced neuropathic pain

**DOI:** 10.1186/1472-6882-13-274

**Published:** 2013-10-21

**Authors:** Vivek Jain, Ashutosh Pareek, Yashumati Ratan Bhardwaj, Nirmal Singh

**Affiliations:** 1 Department of Pharmacy, Banasthali University, Banasthali, Rajasthan 304022, India; 2 Department of Pharmaceutical Sciences and Drug Research, Punjabi University, Patiala-147002, Punjab, India

**Keywords:** *Punica granatum L*, Neuropathic pain, TNF-alpha, BADGE, Oxidative stress

## Abstract

**Background:**

Injury to a nerve is the most common reason of acquired peripheral neuropathy. Therefore, searching for effective substance to recover of nerve after injury is need of present era. The current study investigates the protective potential of Standardized Fruit Extract of *Punica granatum L* (PFE) [Ellagic acid (41.6%), Punicalagins (10%), Granatin (5.1%)] in Tibial & Sural Nerve Transection (TST) induced neuropathic pain in rats.

**Methods:**

TST was performed by sectioning tibial and sural nerve portions of the sciatic nerve and leaving the common peroneal nerve intact. Acetone drop, pin-prick, hot plate, paint brush & Walking Track tests were performed to assess cold allodynia; mechanical heat, hyperalgesia and dynamic mechanical allodynia & tibial functional index respectively. The levels of TNF-α, TBARS, GSH and Nitrite were measured in the sciatic nerve as an index of inflammation & oxidative stress.

**Results:**

TST led to significant development of cold allodynia; mechanical and heat hyperalgesia; dynamic mechanical allodynia; functional deficit in walking along with rise in the levels of TBARS, TNF-α, GSH and Nitrite. Administrations of PFE (100 & 300 mg/kg oral), significantly attenuate TST induced behavioral & biochemical changes. Pretreatments of BADGE (120 mg/kg *IP*) a PPAR-γ antagonist and nitric oxide precursor L-arginine (100 mg/kg *IP*) abolished the protective effect of PFE. Whereas, pretreatment of L-NAME (5 mg/kg *IP*) a NOS inhibitor significantly potentiated PFE’s protective effect of PFE.

**Conclusion:**

PFE shown to have attenuating effect in TST induced neuropathic pain which may be attributed to potential PPAR-gamma agonistic activity, nitric oxide inhibitory, anti-inflammatory and anti oxidative actions.

## Background

According to International Association for the Study of Pain (IASP) Neuropathic pain (NP) is a pain arising as a direct consequence of a lesion or disease affecting the somatosensory system [1]. The prevalence of neuropathic pain is projected at around 10% in the adult population of the US, and is expected to be the similar in other developed countries along with that its prevalence is likely to increase 17% by 2020 countries [2]. NP can crop up from damage to the nerve pathways at any point from cortical neurons in the brain to the terminals of the peripheral nociceptors and symptoms may persist even after the treatment of initial injury [3]. Despite recent advances in the identification of NP the pharmacologic management (Antidepressant, antiepileptics, classical analgesics) of NP remains challenging and yet costly to manage [4]. Therefore, there is a need for new therapies that provide more predictable efficacy in all patients with improved tolerability and it is also important for the clinician to know which drugs are most effective in relieving pain and associated with the fewest adverse effects.

In recent years search shifted towards the substances of natural origin which are prolific source of new drug entity [5]. *Punica granatum L*, commonly known as pomegranate; an ancient, spiritual, and oldest distinctive known edible fruit belongs to the Punicaceae family and is grown in South East Asia, the Mediterranean, the Americas and other parts of the world including India. It is used traditionally as both food and medicine {astringent agent [6] for eradicating parasites [7] along with antipyretic and analgesic in Chinese therapy [8]}. Several tannins, flavonoids, anthocyanidins with various pharmacological and biological activities viz. anti-oxidation [9], anti inflammatory [8], anti diarrhoea, anti-tumour [10], anti-hepatotoxicity [11], anthelmintic, anti-lipoperoxidation [12], anti ulcer [13], anti-bacterial [14], anti viral [15] have been identified in pomegranate. In our own study, hydroalcoholic extract and extract with ellagic acid rich fraction of *Punica granatum L* was documented to attenuate glycerol-induced acute renal failure in rats and dextran sulfate sodium-induced ulcerative colitis respectively [16,17].

Regardless of our present knowledge involving molecular and cellular pathways involved in injury induced neuropathic pain, complete therapeutic prevention or reversal of acute or chronic neuronal injury remains intangible.

However, its potential in neuropathic pain remains to be explored. Therefore, the present study was designed to investigate the ameliorative potential of Punica granatum L in Tibial and Sural nerve transaction induced neuropathic pain in rats.

## Methods

### Experimental animals

Wistar rats of either sex, weighing 200–250 g (procured from NIPER, Mohali) were employed in present study. They were housed in animal cages with free access to water and standard laboratory pellet chow diet (Hindustan Liver Limited, India). The animals with cages were kept in the departmental animal house (Temperature maintained at 18 to 23°C; Humidity maintained at 30 to 35%) and were exposed to normal cycle of light and dark. The experimental protocol was approved by the Banasthali University Institutional Animal Ethics Committee (BUIAEC) and the care of the animals was carried out as per the guidelines of the Committee for the Purpose of Control and Supervision of Experiments on Animals (CPCSEA) (Registration no. 574/02/ab/CPCSEA), Ministry of Environment and Forest, Government of India. After 1 week of acclimatization, rats were used for experimental purpose.

### Drugs and chemicals

Biphenol-A-diglicydyl ether (BADGE) & Gabapentin was purchased from Cayman chemicals. Pomegranate fruit extract (PFE) (Natural Remedies, Bangalore),1,1,3,3 tetra methoxy propane (Sigma Aldrich, USA), DTNB [5,5’-dithio, bis (2-nitro benzoic acid)], BSA (Bovine Serum Albumin) (Sisco Research Laboratories Pvt. Ltd., Mumbai, India), Thio-barbituric acid, L-Arginine (HIMEDIA,India), L-NAME, Folin-Ciocalteu’s phenol reagent (Merck Ltd. Mumbai, India) were procured for the present study. All the reagents used in the present study were of analytical grade.

### Procurement of pomegranate fruit extract (PFE)

Standardized pomegranate fruit extract [Ellagic acid (41.6%), Punicalagins (10%), Granatin (5.1%), heavy metal (Not More Than 10 PPM)] was generous gift from Natural Remedies, Bangalore, INDIA. It was stored in airtight container at low temperature (below 25°C) and suspended in 0.5% carboxy methylcellulose (CMC) before oral administration.

### Induction of neuropathic pain by tibial and sural nerve transection

Peripheral neuropathic pain was induced by tibial and sural nerve transection as described by Dowdall, 2005 [18]. In brief, the rat was deeply anesthetized with ketamine (60 mg/kg *IP*). The skin of its lateral surface of the left thigh was incised and a cut made directly through the biceps femoris muscle to expose the sciatic nerve and its three terminal branches (the sural, common peroneal and tibial nerves). Thereafter, the tibial and sural nerve sections of 2 mm (distal to the trifurcation) were ligated and cut. The common peroneal nerve was left intact and no contact was made with it. The muscle and the skin were closed in two layers. Sham controls were performed by exposing the sciatic nerve and its branches without inducing any lesion. All the surgical procedures were carried out under normal sterile conditions and were performed by the same experimenter.

### Behavioural examination

#### Cold-allodynia (acetone drop test)

The cold allodynia was assessed by spraying a 100 μL of acetone onto the surface of the paw, without touching the skin. The response of the rat to acetone was noted for 20 s and was graded to a 4-point scale as defined by Flatters and Bennett [19]. 0, no response; 1, quick withdrawal, flick or stamp of the paw; 2, prolonged withdrawal or repeated flicking; 3, repeated flicking of the paw with licking of the paw. Acetone was applied thrice to the hind paw, with a gap of 5 min between the acetone applications and the individual scores noted in 20 second interval were added to obtain a single score over a cumulative period of 60 s. The minimum score was 0, while the maximum possible score was 9.

### Mechanical hyperalgesia (pin-prick test)

The mechanical hyperalgesia was assessed by the pin-prick test as described by Erichsen and Blackburn-Munro [20]. The surface of the injured hind paw was touched with the point of the bent gauge needle (at 90° to the syringe) at intensity sufficient to produce a reflex withdrawal response. The duration of the paw withdrawal was recorded in sec.

### Heat-hyperalgesia (hot plate test)

The thermal nociceptive threshold, as an index of thermal hyperalgesia, was assessed by the hot plate, maintained at a temperature of 52.5 ± 1.0°C. The rat was placed on the hot plate and nociceptive threshold, with respect to licking of the hind paw, was recorded in s. The cut-off time of 15 sec was maintained [21].

### Mechanical dynamic allodynia (paint-brush test)

The “paint-brush” behavioural test has been used to explore dynamic responses to a mechanical stimulus. The response to smooth paint-brush has been described as allodynia because normal rats never withdraw from this stimulus. It has been established that dynamic mechanical allodynia is mediated by peripheral low threshold, large myelinated Aβ-fibers [22]. The rat was placed in the cylinder with the wire mesh floor and a smooth paint-brush was used to rub the plantar area of hind paw from the heel to the toes as a stimulus. The stimulus was applied five times with a 5 sec interval and the number of withdrawals was noted (between 0 and 5). The same procedure was repeated twice, with a gap of five min and the total number of withdrawals (in three tests) was added to obtain a single cumulative score of mechanical dynamic allodynia with a minimum value of 0 and maximum of 15 [23].

### Cold-hyperalgesia test (tail immersion test)

Tail cold-hyperalgesia was noted by immersing a terminal part of the tail (1 cm) in the water, maintained at a temperature of 0–4°C. The tail withdrawal latency was recorded and a cut-off time of 20 s was maintained [24].

### Walking track test (De medinacelli method)

Rats were allowed conditioning trials in a walking track (8.2 × 42 cm) darkened at one end. White paper cut to the appropriate dimensions was placed on the bottom of the track. The rat’s hind limbs were dipped in ink, and the rat was permitted to walk down the track, leaving its hind foot prints on the paper [25]. Foot prints were obtained on day 1, 12, and 24 after surgery.

From the footprints, several measurements were undertaken and the so called Tibial Function Index (TFI) was calculated by the equation. TFI of zero defines normal function of the tibial nerve, while a TFI of −100% is presumed to represent complete functional loss.

$$\begin{array}{l} \mathrm{TFI}\;=\;-37.2\left[\frac{\mathrm{EPL}-\mathrm{NPL}}{\mathrm{NPL}}\right]+104.4\;\left[\frac{\mathrm{ETS}-\mathrm{NTS}}{\mathrm{NTS}}\right]\\ \;+45.6\;\left[\frac{\mathrm{EIT}-\mathrm{NIT}}{\mathrm{NIT}}\right]-8.8 \end{array}$$

Print Length (PL) - Distance from the heel to the top of the third toe; Toe Spread (TS) - distance between the first and the fifth toe and Intermediary Toe Spread (IT) represents distance from the second to the fourth toe. These measures were taken both from the non-operated foot [NPL(Non-operated foot Print Length), NTS(Non-operated foot Toe Spread), NIT(Non-operated foot Intermediary Toe Spread)] and from the operated, experimental foot [EPL(Experimental foot Print Length), ETS(Experimental foot Toe Spread) and EIT(Experimental foot Intermediary Toe Spread)].

### Biochemical estimations

The animals were sacrificed after 24th day by high dose anesthesia and the portions of the sciatic nerve and the tissue beneath the sciatic nerve were isolated immediately. The sciatic nerve portion, proximal to the point of transection up to its point of emergence from the spinal cord, and distal to the point of transection up to its ending, was excised. The sciatic nerve homogenate (10%, w/v) was prepared with 0.1 M Tris–HCl buffers (pH 7.4). The tubes with homogenate were kept in the ice water for 30 min and centrifuged at 4°C (2000 × *g*, 10 min). The supernatant of the homogenate was separated and employed to estimate the total protein content and the thio-barbituric acid reactive substances.

### Estimation of protein content

The protein concentration in the sciatic nerve was estimated according to the method of Lowry et al. [26] using bovine serum albumin as a standard.

### Estimation of thio-barbituric acid reactive substances (TBARS)

The estimation of lipid peroxidation was done by measuring the TBARS as described by Okhawa et al. [27]. The absorbance was measured spectrophotometrically at 532 nm. The concentration was expressed in terms of nmol of TBARS/mg of the protein.

### Estimation of reduced glutathione levels

The reduced glutathione levels were measured according to the method of Beutler et al. [28]. The equal quantity of the sciatic nerve homogenate was mixed with tri-chloroacetic acid (10%) and centrifuged to separate the proteins. To 0.01 mL of this supernatant, 2 mL of phosphate buffer (pH 8.4), 0.5 mL of DTNB (5, 5-dithio, bis (2-nitro benzoic acid) and 0.4 mL of double distilled water was added. The mixture was vortexed and the absorbance was taken at 412 nm within 15 min. The concentration of the reduced glutathione was expressed as μg/mg of the protein.

### Estimation of nitrite

Accumulation of nitrite was measured in cell free supernatants by spectrophotometer assay based on Greiss reagent (1% sulphanilamide/0.1% naphthylethylenediamine dihydrochloride/2.5% phosphoric acid) and incubated at room temperature for 10 min to yield a chromophore. Absorbance was read at 543 nm spectrophotometrically. The nitrite concentration was calculated from a standard curve using sodium nitrite as standard and expressed as micro molar nitrite per milliliter homogenate [29].

### Estimation of TNF-α

The sciatic nerve was homogenized in phosphate buffered saline, pH 7.4, and the homogenates were centrifuged at 1500 g, at 4°C for 10 min and the supernatant was immediately used for the TNF-α with commercially available enzyme-linked immunosorbent assay (ELISA). The concentration of TNF-α was expressed in pg/mg of protein [3].

### Experimental protocol

Eighteen groups, each group comprising six Wistar albino rats, were employed in the present study. Behavioral tests were assessed on 1st, 12^th^ and 24th day. Animal were sacrificed on 24^th^ day & the biochemical estimations were done as described earlier. Dose of PFE was selected according to Waghulde et al. [30]. Whole experimental protocol has been shown in Table 1.

**Table 1:** Experimental groups of animals and substances administered

**Groups**	**n**	**Substance**	**Description**
I	6	Normal control	Only fodder and water.
II	6	Sham control	Tibial and Sural nerve exposed without any transaction.
III	6	TST	TST were performed.
IV	6	Vehicle in TST	CMC 0.5% w/v, *p. o.* was administered for 24 days daily.
V	6	PFE *per se*	PFE (300 mg/kg *p. o.* ones daily) was administered to the normal rats for 24 consecutive days.
VI&VII	6	PFE + TST	PFE (100 and 300 mg/kg *p. o.* respectively) were administered to the TST rats for 24 consecutive days
VIII	6	BADGE (PPAR-γ antagonist) + TST	BADGE was administered (120 mg/kg *IP*) to the TST rats for 24 consecutive days.
IX & X	6	BADGE + PFE + TST	BADGE was administered (120 mg/kg *IP*) before PFE (100 and 300 mg/kg *p. o.* respectively) administration to the TST rats for 24 consecutive days.
XI	6	L-NAME + TST	L-NAME was administered (5 mg/kg *IP*) to the TST rats for 24 consecutive days.
XII	6	L-arginine + TST	L-arginine was administered (100 mg/kg *IP*) to the TST rats for 24 consecutive days.
XIII & XIV	6	L-NAME + PFE + TST	L-NAME was administered (5 mg/kg *IP*) before PFE (100 and 300 mg/kg *p. o.* respectively) administration to the TST rats for 24 consecutive days.
XV & XVI	6	L-arginine + PFE + TST	L-arginine was administered (100 mg/kg *IP*) before PFE (100 and 300 mg/kg *p. o.* respectively) administration to the TST rats for 24 consecutive days.
XVII	6	Gabapentin *per se*	Gabapentin (100 mg/kg) *p. o.* administration to the normal rats for 24 consecutive days.
XVIII	6	Gabapentin + TST	Gabapentin (100 mg/kg) *p. o.* administration to the TST rats for 24 consecutive days.

TST- Tibial and Sural Nerve Transection; CMC – carboxymethylcellulose; PFE - Punica granatum L Fruit Extract; BADGE- Biphenol-A-diglicydyl ether; p. o.- per os; IP- Intraperitoneal.

### Statistical analysis

All the results were expressed as mean ± standard error of means (S.E.M.). The data from the behavioral and biochemical results were statistically analyzed by two-way analysis of variance followed by Bonferonni’s post-test and one-way ANOVA followed by Tukey’s multiple range tests, respectively by using Graph pad prism Version-5.0 software. The *P*-value <0.05 was considered to be statistically significant.

## Result

Administration of PFE (300 mg/kg *p. o.*) and GABAPENTIN (100 mg/kg *p. o.*) along with vehicle (carboxymethylcellulose) did not modulate behavioural functions in normal rats.

### Effect of various interventions on tibial and sural nerve transaction (TST)-induced hyperalgesia, allodynia and functional deficit

**Figure 1:**
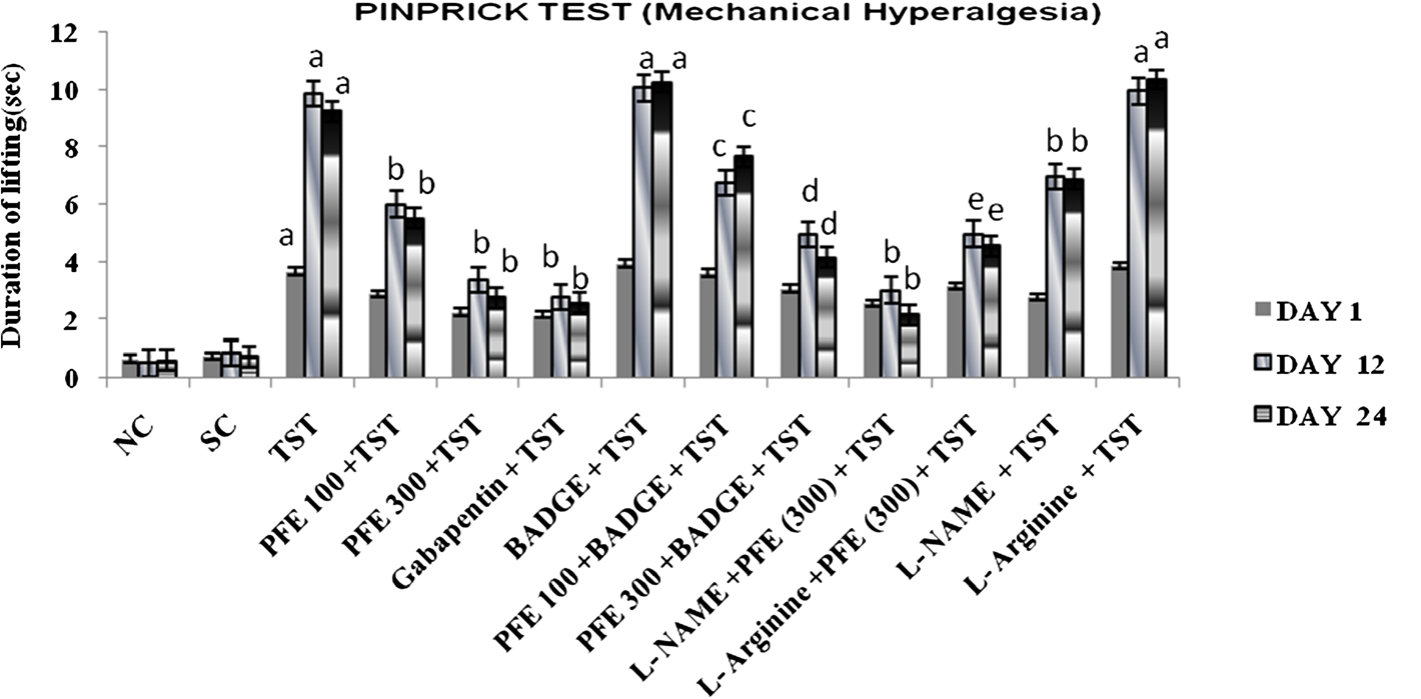
**Effect of PFE on mechanical hyperalgesia, assessed by the pinprick test, in Tibial and Sural nerve Transection (TST)-induced neuropathic pain.** Values are mean ± S.E.M. *n* = 6 rats per group. **a** = *P* < 0.05 vs. sham control group. **b** = *P* < 0.05 vs. TST group. **c** = *P* < 0.05 vs. PFE 100 + TST. **d** = *P* < 0.05 vs. PFE 300 + TST. **e** = *P* < 0.05 vs. L-NAME + PFE (300 mg/kg) + TST.

**Figure 2:**
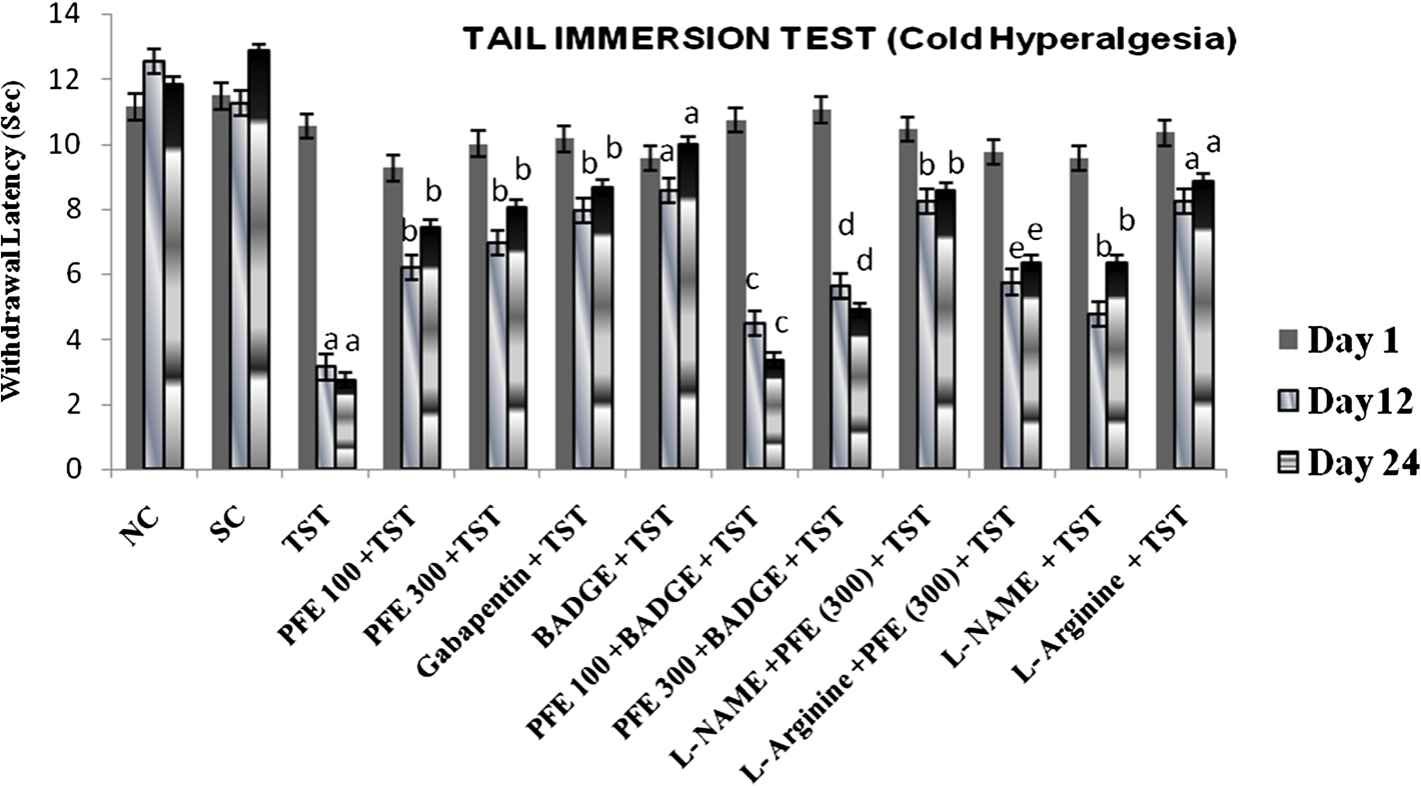
**Effect of PFE on cold hyperalgesia, assessed by the tail immersion test, in Tibial and Sural nerve Transection (TST)-induced neuropathic pain.** Values are mean ± S.E.M. *n* = 6 rats per group. **a** = *P* < 0.05 vs. sham control group. **b** = *P* < 0.05 vs. TST group. **c** = *P* < 0.05 vs. PFE 100 + TST. **d** = *P* < 0.05 vs. PFE 300 + TST. **e** = *P* < 0.05 vs. L-NAME + PFE (300 mg/kg) + TST.

**Figure 3:**
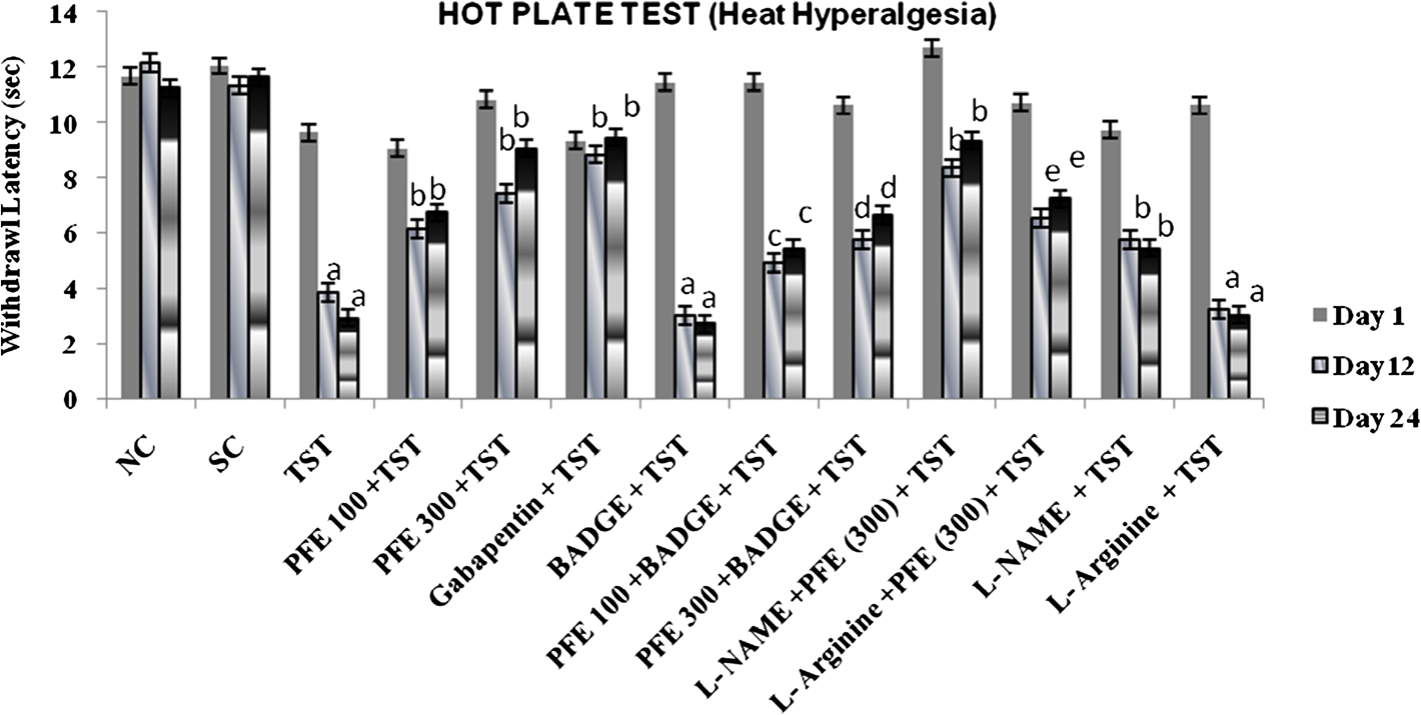
**Effect of PFE on heat hyperalgesia assessed by the hot plate test, in Tibial and Sural nerve Transection (TST)-induced neuropathic pain.** Values are mean ± S.E.M. *n* = 6 rats per group. **a** = *P* < 0.05 vs. sham control group. **b** = *P* < 0.05 vs. TST group. **c** = *P* < 0.05 vs. PFE 100 + TST. **d** = *P* < 0.05 vs. PFE 300 + TST. **e** = *P* < 0.05 vs. L-NAME + PFE (300 mg/kg) + TST.

**Figure 4:**
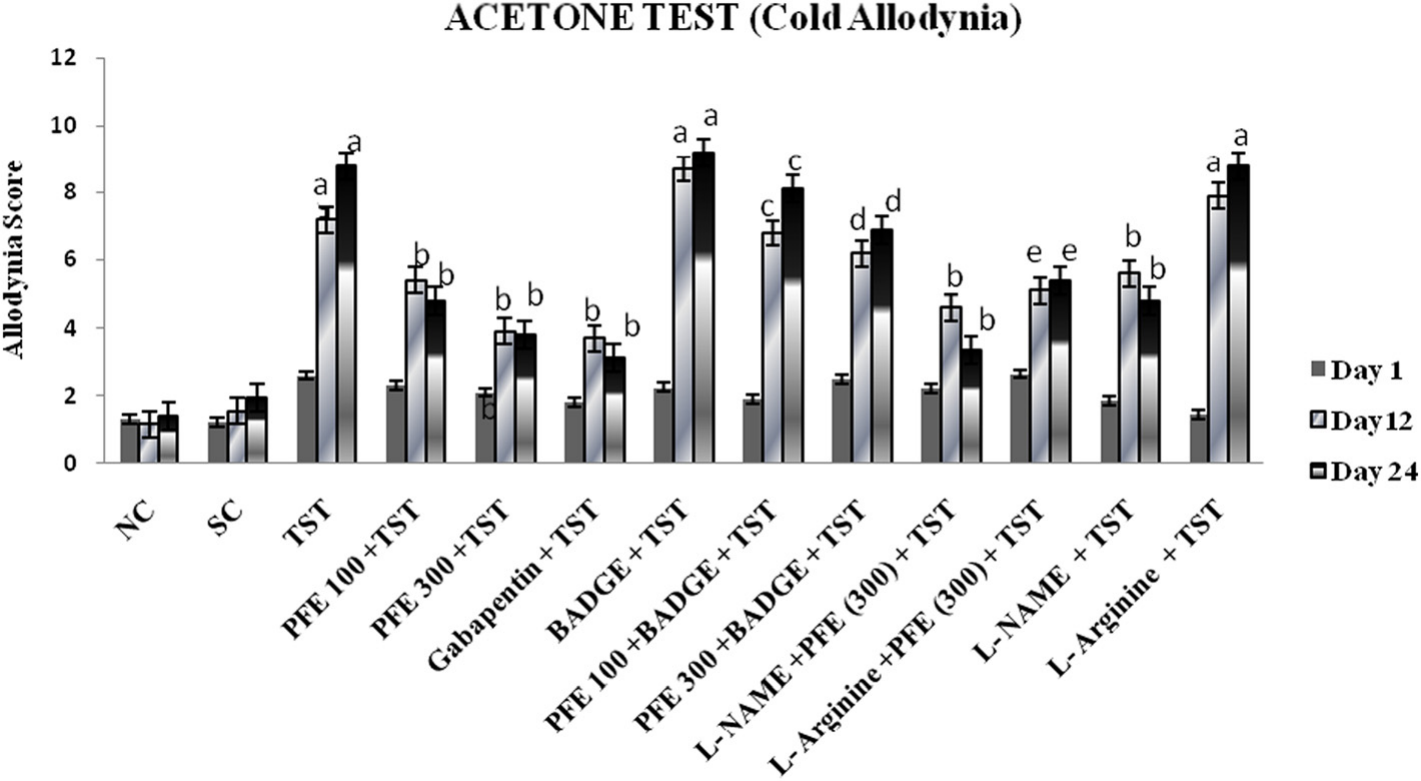
**Effect of PFE on cold Allodynia assessed by the acetone test, in Tibial and Sural nerve Transection (TST)-induced neuropathic pain.** Values are mean ± S.E.M. *n* = 6 rats per group. **a** = *P* < 0.05 vs. sham control group. **b** = *P* < 0.05 vs. TST group. **c** = *P* < 0.05 vs. PFE 100 + TST. **d** = *P* < 0.05 vs. PFE 300 + TST. **e** = *P* < 0.05 vs. L-NAME + PFE (300 mg/kg) + TST.

**Figure 5:**
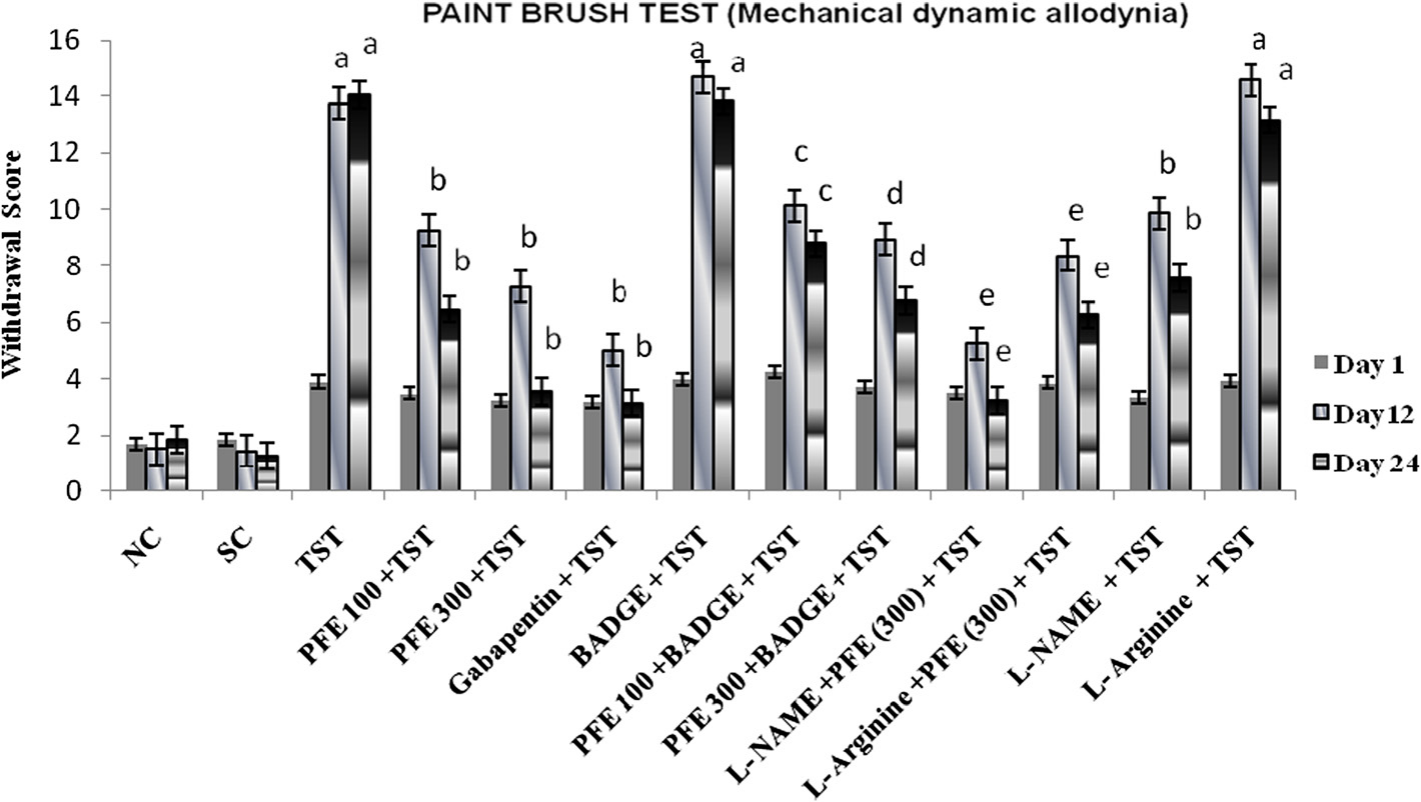
**Effect of PFE on mechanical dynamic Allodynia assessed by the paint brush test, in Tibial and Sural nerve Transection (TST)-induced neuropathic pain.** Values are mean ± S.E.M. *n* = 6 rats per group. **a**= *P* < 0.05 vs. sham control group. **b** = *P* < 0.05 vs. TST group. **c** = *P* < 0.05 vs. PFE 100 + TST. **d** = *P* < 0.05 vs. PFE 300 + TST. **e** = *P* < 0.05 vs. L-NAME + PFE (300 mg/kg) + TST.

TST resulted in significant development of mechanical hyperalgesia (Figure 1); cold hyperalgesia (Figure 2), heat hyperalgesia (Figure 3), cold allodynia (Figure 4), mechanical dynamic allodynia (Figure 5) and functional deficit [in terms of Tibial Functional Index (TFI) {Table 2}] as compared to sham group, as assessed by employing pin prick, tail immersion test, hot plate tests, acetone drop, paint brush test and walking track test respectively. Administration of PFE (100 & 300 mg/kg, *p. o.*) & gabapentin (100 mg/kg *p. o.*) for 24 days attenuated TST -induced hyperalgesia [Cold hyperalgesia {F (22,180) = 18.41}; Heat hyperalgesia {F (22,180) = 52.65}; Mechanical hyperalgesia {F (22,180) = 45.08}], allodynia [Cold allodynia {F (22,180) = 38.40}; Mechanical dynamic allodynia {F (22,180) = 111.40}] and tibial functional deficit (TFI) [F (22,180) = 51.94] in a significant manner. Further, pretreatment of L-NAME (5 mg/kg *IP*) significantly potentiated antihyperalgesic, antiallodynic effect and functional recovery (TFI) of PFE as compared to TST group. In addition, pretreatments of L-arginine (100 mg/kg *IP*) and BADGE (120 mg/kg *IP*) abolished the effect of PFE (100 & 300 mg/kg p.o*.*) on TST-induced hyperalgesia, allodynia and functional recovery (TFI).

**Table 2:** TFI (Walking Track Test): tibial functional index in terms of % functional deficit of nerve in different groups

**Group name**	**Day 1**	**Day 12**	**Day 24**
Normal control	−10.23	−7.24	−11.57
Sham control	−11.54	−6.52	−12.56
Vehicle in TST	−66.15^a^	−88.63^a^	−87.22^a^
TST	−67.45^a^	−93.34^a^	−89.45^a^
PFE 300 mg/kg *per se*	−10.45	−8.12	−12.15
GABAPENTIN100mg/kg *per se*	−11.95	−13.59	−13.78
PFE 100 mg/kg + TST	−58.34^b^	−48.23^b^	−41.16^b^
PFE 300 mg/kg + TST	−54.53^b^	−24.33^b^	−18.54^b^
BADGE + PFE 100 mg/kg + TST	−62.32^c^	−64.22^c^	−67.59^c^
BADGE + PFE 300 mg/kg + TST	−61.13^d^	−58.43^d^	−54.22^d^
BADGE + TST	−69.44^a^	−95.24 ^a^	−90.03^a^
L-NAME + TST	−60.71^b^	−36.25^b^	−31.33^b^
L-arginine + TST	−68.33^a^	−92.28^a^	−88.44^a^
L-NAME + PFE 100 mg/kg + TST	−55.32^b^	−41.79^b^	−37.66^b^
L-NAME + PFE 300 mg/kg + TST	−50.58^b^	−18.56^b^	−15.46^b^
L-arginine + PFE 100 mg/kg + TST	−62.21^a^	−56.42^a^	−49.13^a^
L-arginine + PFE 300 mg/kg + TST	−58.88^e^	−44.78^e^	−41.36^e^
GABAPENTIN 100 mg/kg + TST	−52.21^b^	−20.02^b^	−15.43^b^

a = *P* < 0.05 vs. sham control group.

b = *P* < 0.05 vs. TST group.

c = *P* < 0.05 vs. PFE 100 + TST.

d = *P* < 0.05 vs. PFE 300 + TST.

e = *P* < 0.05 vs. L-NAME + PFE (300 mg/kg) + TST.

### Effect of various interventions on TBARS levels

TST resulted in a significant increase in the levels of thio-barbituric acid reactive substances as compared to sham control (Table 3). Administration of PFE (both doses) for 24 days attenuated TST induces increase in the levels of thio-barbituric acid reactive substances in significant manner [with F (15, 80) =62.10]. Further, L-NAME also attenuated TST induced rise in the levels of TBARS, whereas L-arginine augmented TST induce increase in the levels of TBARS. However, administration of gabapentin & BADGE did not modulate TBARS level in TST induced neuropathic pain.

**Table 3:** Total protein, TBARS, GSH, Nitrite & TNF α level in different groups

**Groups**	**Total protein *(mg/ml)***	**TBARS *(nmol/mg of protein)***	**GSH (μg/mg of protein)**	**Nitrite (μg/ml)**	**TNF α level (*pg/mg of protein)***
Normal control	1.94 ± 0.25	2.87 ± 0.11	50.47 ± 3.21	26.35 ± 2.23	13.34 ± 1.54
Sham control	2.06 ± 0.34	3.07 ± 0.43	51.24 ± 3.05	28.23 ± 2.89	14.03 ±1.06
Vehicle in TST	2.13 ± 0.32	8.97 ± 0.41	27.56 ± 2.46	43.24 ± 2.56	66.14 ± 1.62
TST	2.44 ± 0.24	9.07 ±0.41^a^	28.14 ± 3.05^a^	44.04 ± 2.72^a^	67.11 ± 2.82^a^
PFE 300 mg/kg *per se*	2.11 ± 0.12	3.27 ± 0.12	50.22 ± 2.86	27.13 ± 1.87	12.44 ± 1.35
GABAPENTIN 100 mg/kg *per se*	2.17 ± 0.25	2.84 ± 0.49	50.66 ± 2.45	27.05 ± 1.43	12.57 ± 1.68
PFE 100 mg/kg + TST	2.67 ± 0.46	5.04 ± 0.53^b^	40.12 ± 2.55^b^	37.62 ± 2.54^b^	50.21 ± 2.51^b^
PFE 300 mg/kg + TST	2.21 ± 0.22	3.87 ± 0.38^b^	46.62 ± 2.33^b^	33.15 ± 2.39^b^	21.39 ±2.23^b^
BADGE + PFE 100 mg/kg + TST	2.49 ± 0.32	6.13 ± 0. 36^c^	33.61 ± 2.38^c^	38.18 ± 2.61^c^	57.62 ± 1.11^c^
BADGE + PFE 300 mg/kg + TST	2.16 ± 0.61	5.55 ± 0. 18^d^	38.12 ± 1.58^d^	35.16 ± 2.47^d^	38.01 ± 2.14^d^
BADGE + TST	2.43 ± 0.28	9.87 ± 0. 44^a^	54.67 ± 2.39^a^	46.86 ± 2.77^a^	68.41 ± 2.11^a^
L-NAME + TST	2.10 ± 0.53	6.95 ± 0. 14^b^	37.22 ± 3.04^b^	32.35 ± 2.23^b^	64.17 ± 2.22^b^
L-arginine + TST	2.58 ± 0.23	9.95 ± 0. 46^a^	25.28 ± 2.88^a^	48.61 ± 1.58^a^	66.31 ± 2.35^a^
L-NAME + PFE 100 mg/kg + TST	2.73 ± 0.18	5.01 ± 0.47^b^	39.17 ± 2.23^b^	35.96 ± 2.83^b^	51.89 ± 1.18^b^
L-NAME + PFE 300 mg/kg + TST	2.89 ± 0.29	3.75 ± 0. 23^b^	48.01 ± 1.06^b^	31.48 ± 2.98^b^	21.98 ± 2.21^b^
L-arginine + PFE 100 mg/kg + TST	2.93 ± 0.41	6.14 ± 0.11^b^	37.33 ± 2.81^b^	42.48 ± 2.14^b^	49.47 ± 2.36^b^
L-arginine + PFE 300 mg/kg + TST	2.34 ± 0.45	6.43 ± 0. 59^e^	36.83 ± 2.91^e^	35.36 ± 2.37^e^	22.08 ± 2.38^e^
GABAPENTIN 100 mg/kg + TST	2.09 ± 0.59	8.94 ± 0. 25	31.44 ± 2.33	30.15 ± 3.44^b^	62.16 ± 2.59

a = *P* < 0.05 vs. sham control group.

b = *P* < 0.05 vs. TST group.

c = *P* < 0.05 vs. PFE 100 + TST.

d = *P* < 0.05 vs. PFE 300 + TST.

e = *P* < 0.05 vs. L-NAME + PFE (300 mg/kg) + TST.

### Effect of various interventions on reduced glutathione levels

TST resulted in a significant decrease in the levels of reduced glutathione as compared to sham control (Table 3). Administration of PFE (both doses) for 24 days attenuated TST induces decrease in the levels of reduced glutathione in significant manner [with F (15, 80) =14.07]. Further, L-NAME also attenuated TST induce decrease in the levels of reduced glutathione; whereas L-arginine augmented TST induce decrease in the levels of reduced glutathione. However, administration of gabapentin & BADGE did not modulate reduced glutathione level in TST induced neuropathic pain.

### Effect of various interventions on nitrite level

TST resulted in a significant increase in the levels of Nitrite as compared to sham control (Table 3). Administration of PFE, L-NAME and gabapentin for 24 days attenuated TST induce increase in the levels of Nitrite in significant manner [with F (15, 80) =8.36]. Further, L-arginine augmented TST induce increase in the levels of Nitrite. However, administration of BADGE did not modulate Nitrite level in TST induced neuropathic pain.

### Effect of various interventions on TNF –α level

TST resulted in a significant increase in the levels TNF –α as compared to sham control (Table 3). Administration of PFE for 24 days attenuated TST induces increase in the levels TNF –α in a significant manner [with F (15, 80) =130.0]. However, administration of gabapentin L-NAME, L-arginine & BADGE did not modulate TNF –α level in TST induced neuropathic pain.

## Discussion

Several studies demonstrated that various transaction, ligation, and crushing of sciatic nerve and its branches induces an ipsilateral mechanical, cold, heat hyperalgesia and allodynia thereby indicating the induction of peripheral neuropathic pain [3,31]. These behavioral alterations are constantly present and last over 2–3 weeks, but their time course varying upon the model and species [32]. In the present study Tibial and sural nerve transection-induced alterations in the nociceptive threshold, are consistent with the earlier reports [21,31]. Additionally TST also led to functional deficit in experimental animals. In addition to above behavioral alteration TST also resulted in rise in nerve tissue TNF –α, TBARS and Nitrite levels and fall in reduced glutathione level. The involvement of TNF-α has been well recognized in peripheral as well central sensitization in neuropathic pain [33]. The up-regulation of TNF-α in peripheral nerve region is followed by augment in the cytokine levels in the neurons of dorsal root ganglia of the spinal cord [34]. Further, Data from previous reports confirmed the role of oxidative stress in development of neuropathic pain including TST induced neuropathy [35,36]. Therefore inflammation & oxidative stress play a vital role in TST induced neuropathic pain.

In our study, administrations of PFE (100 & 300 mg/kg *p. o.*) for 24 days significantly attenuate TST-induced behavioral alterations including cold, mechanical and heat hyperalgesia; dynamic mechanical allodynia & cold allodynia. PFE also attenuated the tibial functional index (TFI) in significant manner. In addition, treatment of PFE also prevent TST induced rise in nerve tissue TNF –α, TBARS levels. Moreover, TST induced depletion of reduced glutathione was reversed by PFE treatment. Several studies evidenced that PFE attenuate Alzheimer’s disease [37]. PFE also shown to exert anti-inflammatory activity in several *in vitro* studies [8,38]. Studies have also reported significant anti oxidative effect of PFE [9]. Therefore, it may be specified that the observed ameliorative effect of PFE in TST induced neuropathic pain are probably mediated through its anti inflammatory & antioxidant actions.

Further, it has also been documented that PFE is an activator of PPAR gamma [39]. The presence of PPAR γ receptors in the dorsal horn of the spinal cord indicates their possible involvement in a pain perception and a transmission pathway [40,41]. PPAR γ activators have been documented to attenuate the TST, L5 spinal nerve transection and partial sciatic nerve ligation induced neuropathic pain [9,42]. In this study we observed that pretreatment of BADGE (a PPAR γ antagonist) abolish the protective effect of PFE on TST induced neuropathy. Thereby indicate the involvement of PPAR γ mediated action of PFE in TST induced neuropathy.

On the other hand TST resulted in increase in nitrite level. These findings are in line with a report of injured sciatic nerve of old rats [43]. Earlier reports demonstrated that L5-6 spinal nerves ligation up-regulate the concentration of NOS in the corresponding spinal segments [44]. Augmentation of nitric oxide delayed the schwann cell differentiation consequently slows down functional recovery and axon regeneration after nerve Injury [45]. However, it’s (all three type of NOS) exact role is not clear. In the present study PFE pretreatment significantly attenuated the increase nitric oxide level in TST induced neuropathic pain. Chia-Jung Lee et al. also demonstrated inhibition of NO production and iNOS expression in RAW 264.7 cells by four pomegranate fruit component (Punicalagin, Punicalin, Strictinin A and Granatin B). Moreover, L-NAME (nonselective NOS –inhibitor) and L- Arginine (NO -donor) pretreatment significantly modified the protective effect of PFE, indicating the role of NO pathway in its protective effects. Inhibition of NO pathway by L-NAME significantly potentiates the protective effect of PFE. However, treatment with L-arginine along with PFE significantly abolishes the protective effect of PFE as compared to PFE alone. These finding suggest the possible involvement of NO pathway in protective effect of PFE.

The recent studies have reported that pioglitazone (PPAR gamma agonist) protects dopaminergic neurons against LPS insult at least via inhibiting iNOS expression and NO generation (through PI3K pathway activation), which is potentially mediated via inhibition of p38 MAPK activity [46]. Thus it may be possible that these two signaling pathways play a crucial role in attenuating effect of PFE in TST induced neuropathic pain in an interdependent manner.

Hence the results of the current study, give the impression that, TST-induced nociceptive alterations may be due to the dysregulation of PPAR gamma receptor activity along with increase in inflammation, oxidative stress and nitric oxide production. Therefore, it may be proposed that PFE mediated PPAR gamma agonistic activity, Nitric oxide inhibition, anti-inflammatory activity and decrease in the oxidative stress is responsible for its ameliorative potential in TST-induced neuropathic pain.

Gabapentin is standard drug for neuropathic pain. Therefore, gabapentin was used as positive standard to compare protective effect of PFE in this study.

## Conclusion

Administration of PFE attenuates pain related pain behaviour in Tibial & Sural Nerve Transection injury model. The attenuating effect of PFE in TST may be due to its PPAR gamma agonistic activity, anti-inflammatory properties, nitric oxide inhibition and ability to decrease oxidative stress. Nevertheless, further studies are warranted to explore full potential & mechanism of action of PFE in neuropathic pain.

## Competing interests

The authors declare that they have no competing interests.

## Authors’ contributions

VJ: Contributed in conducting of the study, data collection, data analysis and manuscript preparation. AP: helped in data collection and data analysis. YR: helped in data collection. NS: Contributed in study design, data analysis, and manuscript preparation. All authors read and approved the final manuscript.

## Pre-publication history

The pre-publication history for this paper can be accessed here:

http://www.biomedcentral.com/1472-6882/13/274/prepub
